# eDNA-Mediated Cutaneous Protection Against UVB Damage Conferred by Staphylococcal Epidermal Colonization

**DOI:** 10.3390/microorganisms9040788

**Published:** 2021-04-09

**Authors:** Sapir Ron-Doitch, Marina Frušić-Zlotkin, Yoram Soroka, Danielle Duanis-Assaf, Dalit Amar, Ron Kohen, Doron Steinberg

**Affiliations:** 1 Biofilm Research Laboratory, Faculty of Dental Medicine, Institute of Dental Sciences, Hebrew University-Hadassah, Jerusalem 91120, Israel; sapirr@ekmd.huji.ac.il (S.R.-D.); danielle.assaf@mail.huji.ac.il (D.D.-A.); 2 The Myers Skin Research Laboratory, Faculty of Medicine, Institute for Drug Research, School of Pharmacy, Hebrew University of Jerusalem, Jerusalem 9103401, Israel; marina.zlotkinfrusic@mail.huji.ac.il (M.F.-Z.); yorams@ekmd.huji.ac.il (Y.S.); ronk@ekmd.huji.ac.il (R.K.); 3 Department of Plastic, Reconstructive and Aesthetic Surgery, Faculty of Medicine, Hebrew University of Jerusalem, Israel, Hadassah Medical Center, Jerusalem 9103401, Israel; dalita@hadassah.org.il

**Keywords:** *Staphylococcus*, skin microbiota, Nrf2–Keap1, UVB protection, biofilm eDNA

## Abstract

The human skin is a lush microbial habitat which is occupied by a wide array of microorganisms. Among the most common inhabitants are *Staphylococcus* spp., namely *Staphylococcus epidermidis* and, in ≈20% of healthy individuals, *Staphylococcus aureus*. Both bacteria have been associated with cutaneous maladies, where they mostly arrange in a biofilm, thus achieving improved surface adhesion and stability. Moreover, our skin is constantly exposed to numerous oxidative environmental stressors, such as UV-irradiation. Thus, skin cells are equipped with an important antioxidant defense mechanism, the Nrf2–Keap1 pathway. In this work, we aimed to explore the morphology of *S. aureus* and *S. epidermidis* as they adhered to healthy human skin and characterize their matrix composition. Furthermore, we hypothesized that the localization of both types of bacteria on a healthy skin surface may provide protective effects against oxidative stressors, such as UV-irradiation. Our results indicate for the first time that *S. aureus* and *S. epidermidis* assume a biofilm-like morphology as they adhere to ex vivo healthy human skin and that the cultures’ extracellular matrix (ECM) is composed of extracellular polysaccharides (EPS) and extracellular DNA (eDNA). Both bacterial cultures, as well as isolated *S. aureus* biofilm eDNA, conferred cutaneous protection against UVB-induced apoptosis. This work emphasized the importance of skin microbiota representatives in the maintenance of a healthy cutaneous redox balance by activating the skin’s natural defense mechanism.

## 1. Introduction 

The human skin is the largest interface of our body with its surroundings and as such, it possesses many important physiological roles [1]. It is also home to a milieu of microorganisms: viruses, bacteria, and fungi, which assemble a distinct ecosystem and greatly affect the cutaneous physiology [2]. Two highly explored positive effects that are attributed to cutaneous microbiota are the competitive exclusion of pathogens and immune modulation and training properties [3]. The majority of the identified bacterial genera in human skin are *Corynebacterium*, *Propionibacterium*, and *Staphylococcus*. Among the *Staphylococcus* genus, one of the most abundant and well-characterized skin commensal is *Staphylococcus epidermidis* [2,4], while *Staphylococcus aureus* is part of the normal cutaneous flora of about 20% of healthy individuals [5]. 

*S. aureus* is a cutaneous inhabitant, which is also notoriously known as a very common opportunistic pathogen. Additionally, it has been linked to several cutaneous diseases, namely, atopic dermatitis [6,7], chronic wounds, and surgery-site infections [8], where it is most likely found in a biofilm-type morphology as part of the condition’s pathogenesis [5,9]. *S. epidermidis* is generally referred to as a nonpathogenic, benign skin commensal; however, more recent studies have also correlated it with increased severity of acne vulgaris [10], surgical implants infections [8,11], and neonatal infections [12], which are all associated with assuming biofilm or invasive lifestyles [13].

Biofilms constitute the primary mode of bacterial attachment to both biological and abiotic surfaces [14]. In a human host, biofilms are comprehensively associated with cutaneous (and other) pathologies and are characterized by increased bacterial virulence and antibiotic treatment resistance [15,16,17]. Biofilms are three-dimensional (3D) structures that are composed of immobilized bacteria (both live and dead) that are entangled in many types and forms of extracellular matrix (ECM) [14,18]. The biofilm formation process has five general stages: initial attachment of planktonic bacteria; strong surface adherence, cellular proliferation, and rapid ECM production; increased complexity of the associating matrix and the production of different types of polymers that contribute to biofilm maturation; further maturation, including the formation of complex 3D structures that are designed for nutrient transport and excretion; the final stage, bacterial dispersal [14]. The ECMs of *S. aureus* and *S. epidermidis* biofilms are generally comprised of extracellular polysaccharides (EPS), or more specifically, polysaccharide intercellular adhesin (PIA), as well as extracellular DNA (eDNA) [15,16,19].

Strong and diverse bacterial communities require a sturdy surface area, high water and nutrient intake, and other preferable conditions. However, these requirements are rarely met atop the human skin: constant exposure to damaging environmental elements [20,21,22] (e.g., solar and UV radiations, wind, air pollution, ozone, and mechanical disruptions), dryness, lower temperatures, and intense immune system and keratinocytes antimicrobial activity [23,24], make the skin a seemingly harsh habitat for proliferating bacteria. Moreover, skin-associated bacteria are also exposed to intense shear forces that are caused by rapid keratinocytes shedding and hygienic washes [25]. Nevertheless, as mentioned above, the human skin houses a stable and lush bacterial community. It is therefore apparent that the bacterial colonization’s morphology and characteristics must be highly adaptive, dynamic, and tightly regulated by both bacterial and cutaneous mechanisms. 

As our body’s outermost layer, the skin is constantly exposed to a wide array of harmful oxidative insults stemming from the above-mentioned environmental elements. These insults ultimately lead to the induction of intracellular oxidative stress [26]. Therefore, skin cells must be equipped with powerful protective mechanisms to avoid detrimental consequences, such as programmed cell death via oxidative-damage-induced apoptosis [21]. One of the most substantial antioxidant defense mechanisms in the skin is the nuclear factor erythroid 2-related factor 2–Kelch ECH associating protein 1 (Nrf2–Keap1) pathway [27]. Surprisingly, although Nrf2–Keap1 is a pivotal defense mechanism that must remain constitutively active and alert, it has no known endogenous activators.

We have recently reported a newly described *S.*-*aureus*- and *S.*-*epidermidis*-derived volatile compound, 3-furaldehyde, which induces this pathway in human keratinocytes [28]. In this, and another recent publication [26], we highlight the potential beneficial effects of other components of the intricate skin–bacteria relationship and molecular crosstalk. Nrf2–Keap1 pathway activation occurs in several stages. First, the accumulation of intracellular electrophiles (e.g., reactive oxygen species (ROS)) leads to the oxidation of the Keap1 protein, which normally acts as an Nrf2 inhibitor. This oxidation leads to the release of an active Nrf2, which translocates into the nucleus, where it binds to its binding site in the DNA, the electrophile response element (EpRE). This binding triggers the induction of dozens of cytoprotective genes and enzymes, which are in charge of resolving oxidative damage and promoting cellular survival [21,29].

In this work, we aimed to explore the morphology of *S. aureus* and *S. epidermidis* as they adhere to healthy human skin and evaluate their possible protective effects. We hypothesized that skin-adhered staphylococci would provide cutaneous protection against exogenous oxidative damage via the Nrf2–Keap1 protective cellular mechanism.

## 2. Materials and Methods

### 2.1. Bacterial, Ex Vivo Human Skin, and Human Keratinocytes Cultures

#### 2.1.1. Bacterial Strains and Culture Conditions

The bacterial strains used in this study were *S. aureus* (American Type Culture Collection (ATCC) 25923), and *S. epidermidis* (ATCC 14990). Both bacteria starters were grown overnight in tryptic soy broth (TSB; Accumedia, Neogen, Lansing, MI, USA) at 37 °C. Then, 24 h before skin infections, 1 mL of each bacterial suspension was washed twice with phosphate-buffered saline (PBS; Biological Industries (BI), Beit HaEmek, Israel) and resuspended in 5 mL of high-glucose Dulbecco’s Modified Eagle’s Medium (4.5 g/L D-glucose in DMEM, BI) supplemented with L-glutamine for another overnight growth in 37 °C. These secondary overnight cultures were diluted to obtain 6 × 10^8^ CFU/mL and used for the skin infection experiments [30]. For the skin infection experiments, the inoculum contained 3 × 10^6^ CFU. This amount was determined to achieve a minimal bacterial load that was non-toxic, yet UVB-protective (see below).

#### 2.1.2. Ex Vivo Human Skin Cultures

Human skin cells for ex vivo cultures were obtained from 20-to-60-year-old healthy individuals who had undergone an elective abdominoplasty. Testing was performed according to the Declaration of Helsinki and approved by the Hadassah University Hospital Ethics Committee, #0029-19-HMO (approved: 14 February 2019). Following the manual removal of subcutaneous fat tissue, the skin samples were cut into squares of approximately 0.5 × 0.5 cm, placed dermal side down in the required culture dishes containing high-glucose DMEM supplemented with 1 mM L-glutamine, 100 U/mL penicillin, and 100 U/mL streptomycin, and incubated at 37 °C in a humidified atmosphere of 5% CO_2_. For the bacterial infection experiments, the antibiotics were omitted from the culture media.

#### 2.1.3. Human Keratinocytes Cell Cultures

Immortalized human keratinocytes (HaCaT cells) were cultured in high-glucose DMEM supplemented with 10% (*v*/*v*) fetal bovine serum (FBS; BI), 1 mM L-glutamine, 100 U/mL penicillin, and 100 U/mL streptomycin. The cultures were maintained in an incubator at 37 °C in a humidified atmosphere of 5% CO_2_.

### 2.2. High-Resolution Scanning Electron Microscopy (HR-SEM) of the Staphylococci-Infected Human Skin

For skin infections, 5 µL (containing 3 × 10^6^ CFU) of starter cultures of either *S. aureus* or *S. epidermidis* were seeded on the air-exposed epidermis of the skin fragments and were incubated for 24 and 48 h at 37 °C. The infected and non-treated (NT) control samples were washed and fixed with a 4% *v*/*v* formaldehyde solution for at least 30 min. Then, the fragments were dehydrated in an ethanol gradient and left overnight to air-dry. Prior to the HR-SEM visualization, fragments were mounted onto a metal stub, exposed to a vacuum, and sputter-coated with gold. The SEM images were obtained using a Sirion XL30 SFEG High-Resolution Scanning Electron Microscope (ThermoFisher Scientific, Waltham, MA, USA).

### 2.3. Confocal Scanning Laser Microscopy (CSLM) Imaging of the Whole-Mount Staphylococci-Infected Skin 

Following a 24 h infection of the ex vivo skin with 3 × 10^6^ CFU of either *S. aureus* or *S. epidermidis*, as well as the sterile NT controls, the skin fragments were gently washed and then co-stained for EPS and eDNA with Concanavalin-A (Con-A, final [C] = 25 µg/mL) Alexa Fluor 647 conjugate (Invitrogen, Carlsbad, CA, USA) [31] and TOTO-1 (final [C] = 2 µM) Iodide 514/533 (ThermoFischer Scientific) [32], respectively. The stained fragments were washed, fixed, and kept in glycerol until they were mounted, epidermal side down, onto a microscope cover slide for visualization using a Nikon spinning disk (Yokogawa W1 disk, 50 µm pinhole) confocal microscope equipped with two SCMOS ZYLA cameras (Nikon Instruments Inc., Melville, NY, USA). Z-stack images were obtained at 5 µm increments and analyzed for 3D reconstruction using NIS elements software (v. 5.3, Nikon).

### 2.4. S. aureus Biofilm eDNA Isolation

The *S. aureus* biofilm was grown in TSB supplemented with 1% *w*/*v* D-glucose in six-well plates for 48 h. Then, the supernatant was discharged and the biofilm was gently washed with PBS and scraped into a collection tube, which was subjected to ice-bath sonication and a subsequent proteinase K treatment (final [C] = 5 µg/mL, 1 h, 37 °C) [33]. The suspension was filtered (0.45 µm pores) and subjected to an eDNA isolation process based on a modified cetyltrimethylammoniumbromide (CTAB) method [34]. Briefly, the disrupted biofilm suspensions were diluted 1:1 (*v*/*v*) with 1% *w*/*v*CTAB in a Tris-EDTA (TE) buffer, incubated at 65 °C for 30 min, and centrifuged. Pellets were resuspended in a high-salt TE buffer (1 M NaCl), diluted with a 0.6 volume of isopropyl alcohol (IPA), and incubated for 1 h on ice. Following centrifugation, the pellets were resuspended in the TE buffer and subjected to the phenol:chloroform:isoamyl alcohol DNA separation method. The eDNA in the achieved supernatants was precipitated with 0.2 M NaCl in 70% *v*/*v* ethanol at −20 °C overnight, then washed twice with cold ethanol, and finally reconstituted in nuclease-free water.

### 2.5. Human Caspase 3 Activity Evaluation Following UVB Irradiation in Ex Vivo Skin

The extent of the UVB-induced apoptosis in the bacteria- and eDNA-pretreated skin was evaluated using the caspase 3 assay [35]. Skin fragments were treated with 3 × 10^6^ CFU of either a *S. aureus* suspension, a *S. epidermidis* suspension, or an isolated eDNA solution for 24 h. The next day, the fragments were washed and irradiated using a UVB source at 300 mJ/cm^2^ and placed in fresh, treatment-free media for an additional 24 h. Finally, the epidermis was separated from the dermis by placing fragments in PBS at 56–60 °C for 1 min and gently peeling the epidermis off using a scalpel. The separated epidermises were individually placed in a 96-well plate containing 2.5 mM Ac-DEVD-AMC (AnaSpec, Inc., Fremont, CA, USA), 0.02% Triton X-100, and 10 mM dithiothreitol at 37 °C. The Fluorescence intensity of the released coumarin derivative was measured at λ_ex_ = 390 nm, λ_em_ = 435 nm. The caspase 3 activity is given using the fluorescence-versus-time slope, which was calculated over 20 min in the linear range, and is expressed as a percentage of the irradiated NT control. In one protocol the skin was co-treated with eDNA and Ochratoxin A (OTA), an Nrf2 translocation inhibitor. A total of 3 µM of OTA (a sub-toxic concentration) dissolved in ethanol was topically applied to the epidermis, followed by the application of eDNA in required concentrations 30 min later, to allow for ethanol evaporation.

### 2.6. Nrf2 Nuclear Translocation Assessment

The nuclear accumulation of Nrf2 in HaCaT cells following the eDNA treatments was assessed using an immunofluorescence (IF) method. The cells were treated for 6 h and then fixed, permeated, and blocked, followed by overnight incubation with primary rabbit anti-human Nrf2 antibody (ab62352, Abcam, Cambridge, U.K.). The next day, a secondary goat anti-rabbit IgG antibody labeled with AlexaFluor®647 (ab150083, Abcam) was added for staining (1 h, RT). The cells were then visualized using a Cytation 3 cell imaging reader (BioTek, Winooski, VT, USA) and the obtained images were analyzed and quantified using ImageJ software (v. 1.53e, National Institutes of Health, Bethesda, MD, USA). The fluorescence intensity of all the images was adjusted to the NT control. Data are expressed as the mean nuclei-to-cytoplasm fluorescence intensity signal ratio (nuc/cyt ratio) in the treated sample minus the same value for the control (NT) sample, i.e., Δ(nuc/cyt). Positive values indicate higher nuclear accumulation in the treated samples relative to the NT control.

### 2.7. Statistical Analysis

All data points represent the results from at least triplicates in at least three biological repeats. All data are presented as the mean ± standard error (SEM) unless otherwise specified. The statistical analysis was conducted using freeware found at https://astatsa.com (accessed on 8 April 2021), where multiple comparisons were performed using ANOVA followed by a post hoc Tukey’s honestly significant difference (HSD) test. Differences were termed statistically significant for *p* < 0.05.

## 3. Results

### 3.1. S. aureus and S. epidermidis Adhered to Ex Vivo Human Skin 

**Figure 1:**
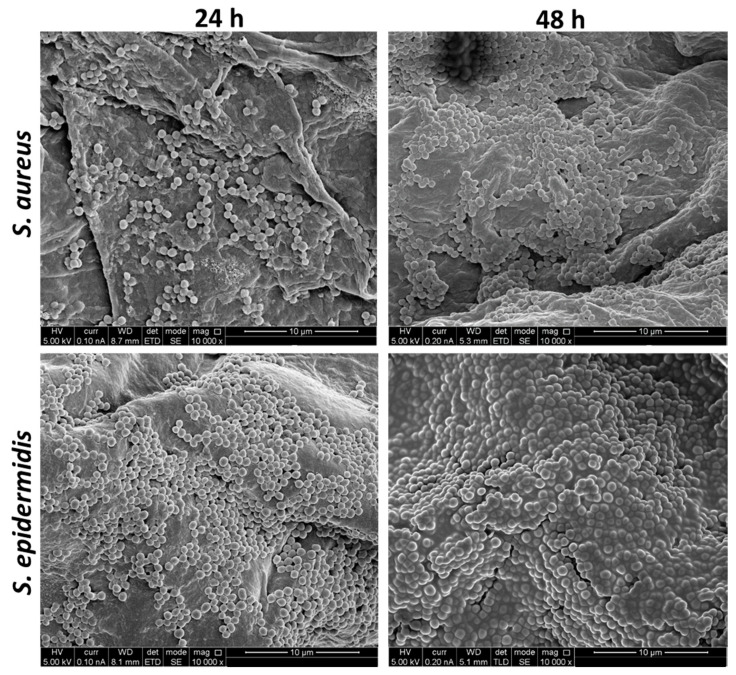
Scanning electron microscopy (SEM) images of ex vivo human skin that was infected with *S. aureus* and *S. epidermidis* for 24 or 48 h. Cocci-shaped bacteria appeared to adhere to the surface and become entangled in a matrix-like substance. Magnification: ×10k.

The SEM images showed many spherical cocci-shaped bacteria that were strongly adhered to the skin fragments’ surfaces (Figure 1). After 48 h, the cocci appeared to be entangled or wrapped in a matrix-like substance (Figure 1, right panels). Noninfected skin appeared sterile (Supplementary Materials Figure S1). The bacteria seemed to accumulate in higher numbers in the folds and crevices of the skin, rather than on smoother surfaces. No significant differences in the cell numbers or matrix association were visible between both tested species (Figure 1, compare top and bottom panels).

### 3.2. Positive Stains for eDNA and EPS in Skin-Adherent S. aureus and S. epidermidis Cultures

**Figure 2:**
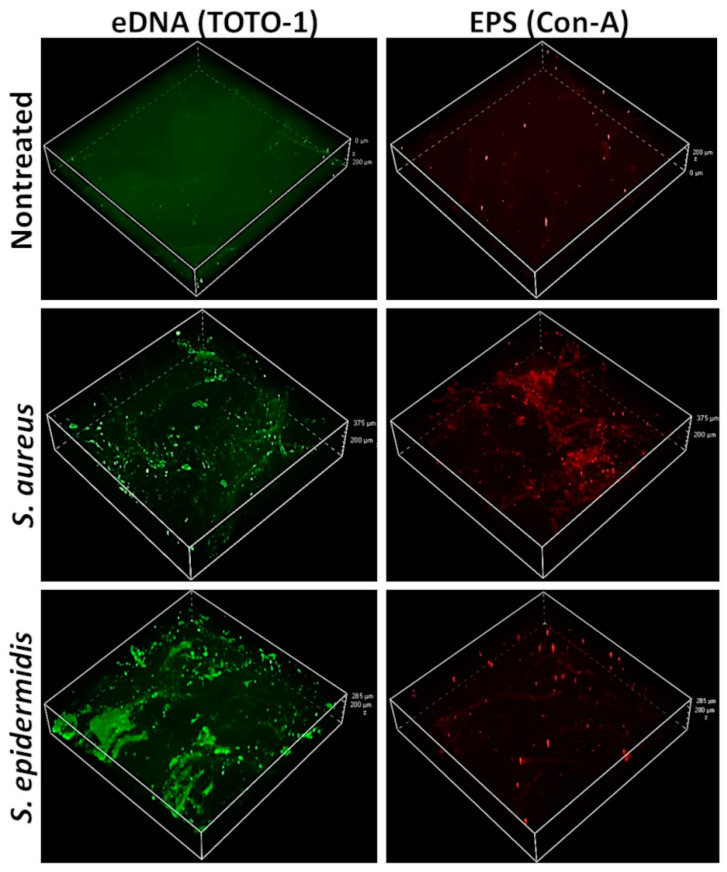
Confocal scanning laser microscopy (CSLM) reconstructed 3D images of ex vivo human skin 24 h post infection with *S. aureus* and *S. epidermidis.* Both types of bacteria adhered to the skin’s surface and created an extracellular matrix (ECM), which stained positive for extracellular DNA (eDNA; TOTO-1, green, left panels) and extracellular polysaccharides (EPS; Concanavalin-A, red, right panels). Top panels: nontreated (NT), sterile skin; middle panels: *S.*-*aureus*-infected skin; bottom panels: *S.*-*epidermidis*-infected skin. Magnification: ×10, the z-axis scale bar is shown for each image.

The reconstructed CSLM images showed that both the *S.*-*aureus*- and *S.*-*epidermidis*-infected ex vivo skin samples stained positively for eDNA (Figure 2, left panels, green, TOTO-1 dye) and EPS (Figure 2, right panels, red, Concanavalin-A dye). The noninfected sterile skin showed a weak green background stain (Figure 2, top left panel) and almost no red background stain (Figure 2, top left panel). The *S. aureus* appeared to produce both eDNA and EPS in similar amounts (Figure 2, middle panels), while the *S. epidermidis* appeared to produce more eDNA than EPS (Figure 2, bottom panels). The image quantification data are presented in Supplementary Materials Figure S2. 

### 3.3. S. aureus and S. epidermidis Pre-Treatment of Human Skin Fragments Provided Protection against UVB-Induced Epidermal Apoptosis

**Figure 3:**
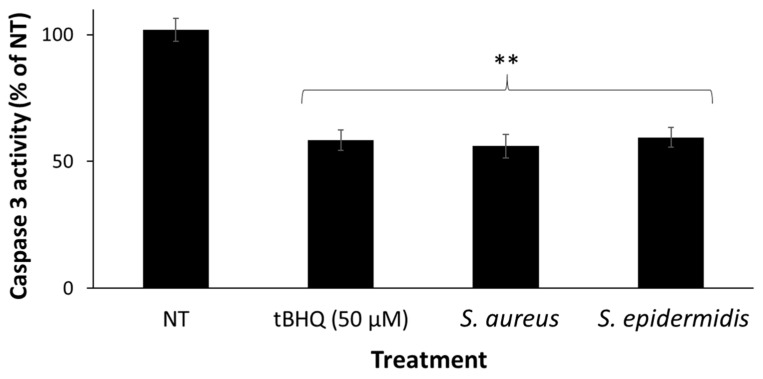
Caspase 3 activity in ex vivo human skin following 24 h of infection with *S. aureus* and *S. epidermidis*, and the consequent irradiation with an apoptosis-inducing UVB source at 300 mJ/cm^2^. Both bacteria caused a significant reduction in the caspase 3 protein activity in the skin. *Tert*-Butylhydroquinone (tBHQ) served as a positive control. The results were normalized to the nontreated control and presented as a percentage of NT. ** *p* < 0.01, treated sample vs. NT.

Caspase 3 activity is a well-known marker for eukaryotic programmed cell death via apoptosis. High-dose UVB irradiation initiates an intracellular signaling cascade that activates, among many other enzymes, the cysteine-aspartate protease caspase 3, ultimately leading to cellular apoptosis. It is shown here that infection of the skin with either *S. aureus* or *S. epidermidis* 24 h before high-dose UVB irradiation led to a significant reduction in caspase 3 activity, indicating protection against UVB-induced apoptosis. *tert*-Butylhydroquinone (tBHQ) served as a positive protective control (reducing caspase 3 activity by 41.6%, *p* < 0.01, compared to the NT irradiated control; Figure 3). *S. aureus* and *S. epidermidis* treatments reduced the cutaneous caspase 3 activity by 44% and 40.5%, respectively (*p* < 0.01, normalized to the NT irradiated control; Figure 3). 

### 3.4. Isolated eDNA from the S. aureus Biofilm Provided Protection against UVB-Induced Epidermal Apoptosis to Ex Vivo Human Skin 

**Figure 4:**
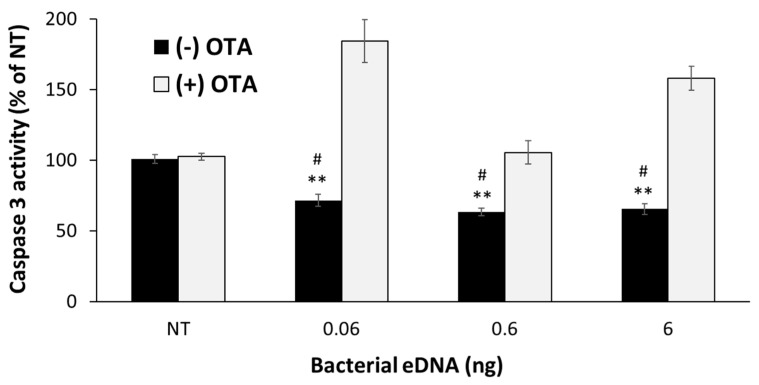
Caspase 3 activity in ex vivo human skin following 24 h of eDNA treatments isolated from *S. aureus* biofilms and subsequent irradiation with a UVB source at 300 mJ/cm^2^ (black bars). The white bars show the caspase 3 activity of the co-treatments of eDNA and ochratoxin A (OTA), an Nrf2 mechanism inhibitor. The eDNA treatments significantly reduced the caspase 3 activity, while the OTA co-treatments led to significantly increased caspase 3 activity. The results were normalized to a nontreated control and presented as a percentage of NT. ** *p* < 0.01, treated sample vs. NT; # *p* < 0.001, (−)OTA vs. (+)OTA.

In a similar experiment to that described above in Section 3.3., epidermal caspase 3 activity was evaluated following pretreatment with eDNA derived from a *S. aureus* biofilm. Skin UVB protection, which is expressed as a reduction in caspase 3 activity levels, is considered to be mediated by the activation of the eukaryotic Nrf2–Keap1 cellular protective pathway. Therefore, the use of an Nrf2 nuclear translocation inhibitor, OTA, would block the ability of a treatment to induce this pathway and consequently lead to a high caspase 3 activity, which would be expressed as a higher rate of UVB-induced damage and apoptosis. It is shown here that all the tested amounts of isolated *S. aureus* biofilm eDNA caused ex vivo human skin to have significantly reduced caspase 3 activity, i.e., protection against UVB apoptotic damage (Figure 4, black bars; 0.06, 0.6, and 6 ng of eDNA reduced caspase 3 activity by 28.6%, 36.9%, and 34.7%, respectively; *p* < 0.01 for eDNA vs. the NT irradiated control). Moreover, the OTA treatment abolished the observed protective effect of the eDNA, leading to a significant increase in the caspase 3 activity in the skin fragments (Figure 4, light gray bars; treatments of 3 µM OTA combined with 0.06, 0.6, and 6 ng of eDNA increased the caspase 3 activity by 112.8%, 42.3%, and 92.5%, respectively; *p* < 0.001 for eDNA (–) OTA vs. (+) OTA). These results strongly indicate an Nrf2–Keap1 involvement in the observed protective effects of the *S. aureus* eDNA on ex vivo human skin. 

### 3.5. eDNA Extracted from an S. aureus Biofilm Activated the Nrf2–Keap1 Cellular Pathway in Human Keratinocytes

**Figure 5:**
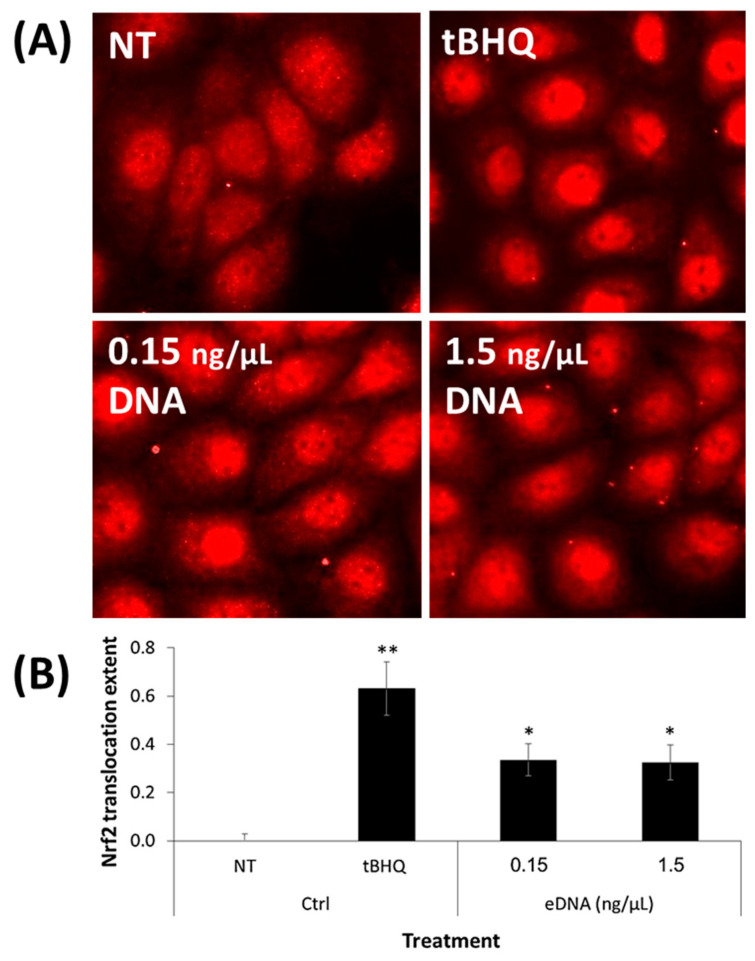
eDNA treatments induced Nrf2 nuclear translocation in HaCaT cells after a 6 h incubation. (**A**) Nuclear translocation of Nrf2 visualized using immunofluorescent staining (Alexa-fluor-647-labeled antibody, red) and imaging (magnification: ×20); (**B**) fluorescent signal quantification using ImageJ software. Data are expressed as the difference in the nuclear-to-cytoplasm fluorescent signal ratio between the treated and nontreated cells (Δnuc/cyt ratio). * *p* < 0.05, ** *p* < 0.01 treated samples vs. NT.

To substantiate the indication of Nrf2–Keap1 mechanism’s involvement in the observed UVB protection granted by eDNA in ex vivo human skin (see Section 3.4. and Figure 4), the activation of this pathway by isolated eDNA was evaluated in a human keratinocytes cell culture (HaCaT cells) using immunofluorescence staining. Since Nrf2 is a nuclear factor, a precondition for the pathway activation would be its nuclear translocation. Indeed, it was shown that a 6 h eDNA treatment led to the nuclear accumulation of immune-labeled Nrf2 protein in human keratinocytes (Figure 5A, bottom panels), which was also found to be statistically significant after semi-quantitative image analysis (*p* < 0.05; Figure 5B; the Δnuc/cyt ratio (NT-treatment, see Section 2.6 for the equation) was 0.34 and 0.33 for 0.15 and 1.5 ng/µL eDNA, respectively). tBHQ served as a positive Nrf2-inducing control, with significant nuclear accumulation (Figure 5A, top-right panel; Δnuc/cyt ratio of 0.63 (*p* < 0.01; Figure 5B)).

## 4. Discussion 

In this work, we aimed to explore the hypothesis that the cutaneous residents *S. aureus* and *S. epidermidis* portray an important role in skin physiology in a novel context, protection against environmental stressors, and specifically UVB exposure as an example. 

In the first stage of the research, we established an ex vivo human skin infection model, i.e., a type of skin–bacteria co-culture, where the first inoculum was set at 3 × 10^6^ CFU. This number roughly correlates with some reports of bacterial amounts present on healthy human skin [36], although these numbers are highly variable, depending on the skin area, sampling technique, and bacterial identification method [37]. The air-exposed epidermal surface was inoculated with either *S. aureus* or *S. epidermidis* and its surface properties were visually assessed after 24 h (and 48 h for SEM experiments) using SEM and CSLM. Both imaging techniques showed bacterial surface adhesion and ECM production (Figure 1 and Figure 2). The CSLM imaging further revealed part of the composition of the visible ECM: EPS and eDNA (Figure 2, positive stains for Con-A and TOTO-1). Staphylococcal EPS are essential for the surface association, and polysaccharide intercellular adhesin (PIA) is a hallmark for solid surface adhesion [17,38]. eDNA is another important component of Staphylococcal biofilms; it is known to contribute to single-cell adhesions and stabilize the structures of young biofilms [17,32].

Biofilms are defined as bacterial populations that adhere to each other and/or to a surface and are surrounded by an ECM [14]. Intriguingly, the topic of matrix composition and morphological properties of cutaneous staphylococci in a healthy, commensal context compared to very well-described pathological states is rarely mentioned in the literature [6,39]. Most publications refer to diseased skin (namely biopsy sample imaging) [40,41], animal or synthetic skin models [42,43,44], or clinical isolates cultured in vitro under laboratory conditions [45,46]. In these and numerous other disease conditions, there is general agreement among the scientific community that the dominant bacterial morphology is the biofilm form. This, of course, is correlated with increased invasiveness, virulence, and treatment resistance [11,38,40,45,46]. The results of this current study suggest that skin-associated *S. aureus* and *S. epidermidis* produce EPS and eDNA, which resemble ECM production, thus indicating that they may also assume a biofilm form while inhabiting healthy human skin. Since the skin’s solid surface is a harsh habitat that is exposed to environmental insults and constant immune system activity, it might explain why the bacteria aggregate together in a community with a more shielded and resistant architecture.

From an evolutionary standpoint, it is presumable that for the skin to tolerate bacterial colonization on its surface, their presence should confer advantageous properties. This is inferred due to the known positive attributes of the cutaneous microbiota on the skin, specifically by immune training and modulation [2,6,25,47]. Hence, the next stage of this research aimed to explore whether the presence of *S. aureus* and *S. epidermidis* on healthy skin provided beneficial effects in the form of improved skin defense against environmental insults. As an example for a common exogenous stressor, the bacteria-treated skin was exposed to UVB irradiation. The assay used for this evaluation derived from the known cutaneous effects of UVB exposure, which is a toxic, apoptosis-inducing oxidative damage. Caspase 3 is an effector caspase, a downstream member of this intricate apoptotic cascade, which is known to be highly activated following a high UVB irradiation dose to skin [35,48]. A protective treatment would therefore lead to decreased caspase 3 activity following UVB irradiation, indicating apoptosis resistance and cellular survival [49]. Indeed, our results show that both *S. aureus* and *S. epidermidis* pretreatments led to significant reductions in caspase 3 activity (Figure 3), i.e., protection against UVB-induced apoptosis. 

This highly interesting result showing very similar protection patterns of both tested bacteria raised a question regarding the mechanism of this conferred apoptosis resistance. One opinion might be that the bacterial layer functions as a physical barrier that filters damaging UVB rays. Under this premise, it should be noted that staphylococci possess many oxidative stress resistance mechanisms, including ROS-detoxifying proteins and membrane lipids oxidation involvement [50,51]. More recent data show that *S. epidermidis* can resist UV damage via glycerol fermentation [52], where a product of this fermentation process, butyric acid, was shown to reduce UVB-induced cutaneous proinflammatory cytokine production in a murine model [53]. These observations may, of course, be part of the contributors to the observed cutaneous UVB protection in the present study. Nevertheless, the new results presented here highlight the involvement of a more subtle, intricate biochemical mechanism that relies on the crosstalk between the skin tissue and its bacterial inhabitants, which is mediated by specific molecular components. When examining the CSLM images and quantification (Figure 2 and Supplementary Materials Figure S2), it was apparent that there were significant differences between both bacteria in terms of ECM excretion: *S. aureus* stained positively and similarly for both EPS and eDNA, while *S. epidermidis* showed a very week EPS stain but a substantial eDNA stain. Therefore, it is less likely that staphylococcal-mediated UVB-protection stems merely from the physical barrier function. In addition to this observation, current research shows that bacterial eDNA can interact and affect human components (namely protein) [54,55]. Put together, these findings pointed to the possible involvement of staphylococcal eDNA in the observed skin protection by *S. aureus* and *S. epidermidis*. 

Indeed, when testing the eDNA that was derived from isolated *S. aureus* in a similar setting of UVB protection assessment via caspase 3 activity assay, it became apparent that staphylococcal eDNA was in fact an important factor in cutaneous UVB protection, leading to a significant decrease in caspase 3 activity (Figure 4). The tested amounts of eDNA were chosen in correlation with known quantities found in *Staphylococcus* biofilms [56,57], while also covering a wide (threefold) range of concentrations. Moreover, in this experimental setting, we also introduced OTA, an Nrf2–Keap1 pathway inhibitor, to evaluate whether the eDNA protection was mediated via this important cellular mechanism. Our results show that Nrf2–Keap1 inhibition abolished the observed protection granted by the eDNA pretreatment, strongly indicating Nrf2’s involvement in this process.

Finally, we used a human keratinocyte model to confirm that *S. aureus* eDNA initiates Nrf2 nuclear translocation, the major step in the Nrf2–Keap1 pathway activation. As shown in Figure 5, the labeled Nrf2 significantly accumulated in the cell nuclei, corroborating the ex vivo skin model’s response to OTA. 

The underlying molecular mechanism by which *S. aureus* and *S. epidermidis* cultures, as well as isolated bacterial eDNA, activate the Nrf2–Keap1 protective cutaneous pathway remains unclear and requires further investigation. Nevertheless, we have recently published an opinion letter [26] stating that the skin might sense the localization of commensal bacteria and their excreted eDNA on its surface as a form of moderate, beneficial stress. Furthermore, we postulate that each stress applied to the skin is translated downstream into oxidative stress, namely intracellular ROS augmentation. These, in turn, may act as electrophiles and activate the Nrf2–Keap1 mechanism, thus leading to increased cutaneous resistance to UVB-induced damage via the evasion and resolution of the apoptotic cascade. 

## 5. Conclusions 

*S. aureus*, a notoriously famous opportunistic pathogen, and *S. epidermidis*, a usually harmless commensal bacteria which is nevertheless linked to certain pathologies, are presented here in a new light. Their advantageous and beneficial effects on the human skin were highlighted, as they were shown to confer protection against UVB-induced cellular damage. Moreover, both bacteria’s healthy skin colonization morphology was initially described, revealing a biofilm-like surface association technique by utilizing known ECM material, EPS, and eDNA. The eDNA component of skin-adhered *S. aureus* and *S. epidermidis* cultures was shown to be of high significance to the conferred cutaneous UVB protection, most likely via the activation of the important cell-protecting Nrf2–Keap1 pathway. Thus, together with our recent observation regarding the protective effect of a volatile bacterial metabolite, namely, 3-FA [28], it is becoming apparent that these common human skin colonizers are substantial factors in the maintenance of a healthy cutaneous redox balance, as they emerge as natural Nrf2–Keap1 pathway activators. Finally, it is also possible to hypothesize that a positive-feedback-type mechanism is in play: the unforgiving nature of the skin’s surface encourages its resident bacteria to assume a biofilm-type morphology, thus leading to increased eDNA production, which in turn confers protection against an environmental insult.

## Data Availability

Not applicable.

## Supplementary Materials

The following are available online at https://www.mdpi.com/article/10.3390/microorganisms9040788/s1, Figure S1: SEM images of ex-vivo human skin fragment; Figure S2: Quantification analysis of CSLM images of *S. aureus* and *S. epidermidis* infected skin fragments.
