# Supplementary material for: eDNA-Mediated Cutaneous Protection Against UVB Damage Conferred by Staphylococcal Epidermal Colonization
Source: Microorganisms. 2021 Apr 9;9(4):788. doi: 10.3390/microorganisms9040788 (PMC8068790; doi:10.3390/microorganisms9040788)
Supplement: Supplementary file 1 [file microorganisms-09-00788-s001.pdf]

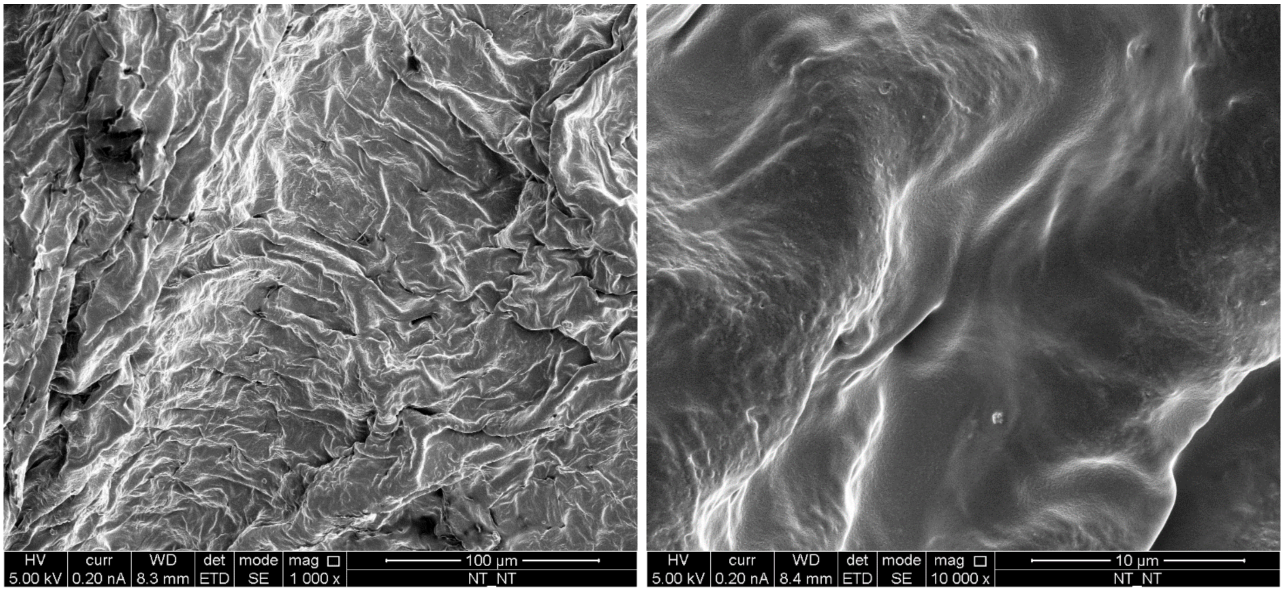

Figure S1. SEM images of *ex-vivo* human skin fragment. Non infected (NT) samples appear sterile, no bacteria are present on the surface. Magnification: left panel X1000; right panel X10k

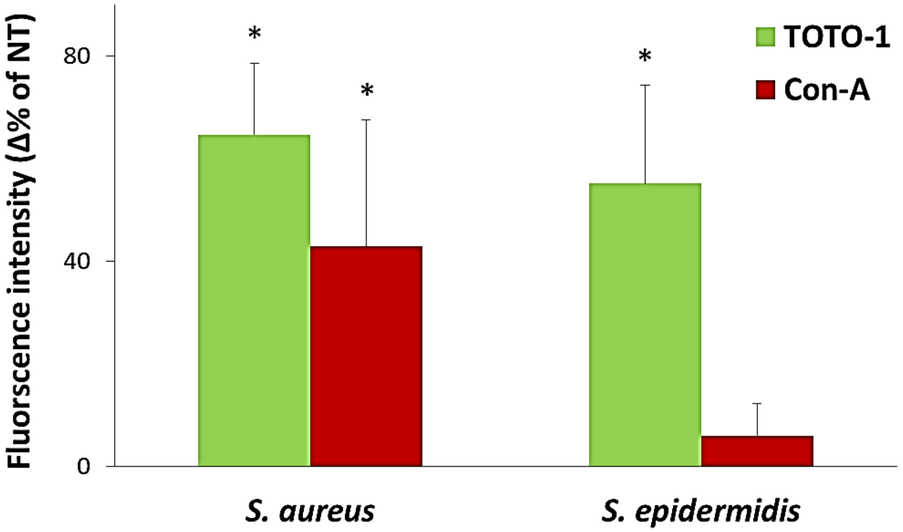

Figure S2. Quantification analysis of CSLM images of *S. aureus* and *S. epidermidis* infected skin fragments (24 h treatments) and stained for eDNA (TOTO-1 dye, green bars) and EPS (Con-A dye, red bars). Non-treated (NT) fragments show non-specific background stains and were set as reference. Treated samples are normalized to NT non-specific stain. Data are expressed as the delta of treated-NT normalized values (Δ % of NT). *p<0.05, treated vs. NT samples.
